# Establishing a core outcome set for use in trials of severe acute paediatric asthma exacerbation (2–17 years): study protocol for an international co-design Delphi survey – a Paediatric Emergency Research Network study initiative

**DOI:** 10.1136/bmjopen-2026-124611

**Published:** 2026-10-29

**Authors:** Charmaine Gray, Leah Armit, Franz E Babl, Colin Powell, Adriana Yock-Corrales, Indumathy Santhanam, Michael D Johnson, Stuart Dalziel, Gene Ong Yong-Kwang, Morgan M Millar, Suzanna Schuh, Jose Irazuzta, Sebastián González, Lilian Chebet Kimeto, Janvier Hitayezu, Tigist Bacha, Abate Yeshidinber, Raghavendra Vanaki, Romit Saxena, Hasitha Gajaweera, Mohammod Jobayer Chisti, Damian Roland, Ian Maconochie, Jordan Evans, Khalid Alansari, Abdullah Ghanem, Saboora Waris, Ayeesha Saeed, Carlos Ramon Iglesias, Dorine Borensztajn, Johan N Siebert, Simon Craig, Pediatric Emergency Research Networks (PERN) asthma working group, Rosie Rock

**Affiliations:** 1 Emergency Department, Flinders Medical Centre, Bedford Park, South Australia, Australia; 2 College of Medicine and Public Health, Flinders University, Adelaide, South Australia, Australia; 3 Departments International Collaborative, PREDICT Paediatric Research in Emergency, Melbourne, Victoria, Australia; 4 Emergency Department, The Royal Children’s Hospital Melbourne, Melbourne, Victoria, Australia; 5 Murdoch Children’s Research Institute, Clinical Sciences, Parkville, Victoria, Australia; 6 Department of Paediatrics and Department of Critical Care, The University of Melbourne, Parkville, Victoria, Australia; 7 School of Medicine, Cardiff University, Cardiff, UK; 8 National Children’s Hospital, San Jose, Costa Rica; 9 University of Costa Rica, San Jose, Costa Rica; 10 Regional Collaborative Center, Institute of Child Health, Madras Medical College, Chennai, India; 11 Pediatric Resuscitation and Emergency Research Network (PRERNI), India, India; 12 Primary Children’s Hospital, Salt Lake City, Utah, USA; 13 Department of Pediatrics, The University of Utah School of Medicine, Salt Lake City, Utah, USA; 14 Pediatric Emergency Care Applied Research Network, Salt Lake City, Utah, USA; 15 Departments of Surgery and Paediatrics: Child and Youth Health, The University of Auckland, Auckland, New Zealand; 16 Children’s Emergency Department, Starship Children’s Health, Auckland, New Zealand; 17 Department of Emergency Medicine, KK Women’s and Children’s Hospital, Singapore; 18 University of Utah School of Medicine, Salt Lake City, Utah, USA; 19 Division of Paediatric Emergency Medicine and Research Institute, Hospital for Sick Children Research Institute, Toronto, Ontario, Canada; 20 University of Florida, Gainesville, Florida, USA; 21 Red Colaborativa Pediatrica de Latinoamercia, Montevideo, Uruguay; 22 Unidad de Cuidados Intensivos de Niños del Centro Hospitalario Pereira Rossell, Facultad de Medicina, Universidad de la Republica, Montevideo, Uruguay; 23 Department of Paediatrics and Child Health, Kenyatta University, Nairobi, Kenya; 24 Mater Misericordiae Hospital, Nairobi, Kenya; 25 Centre Hospitalier Universitaire de Kigali, Kigali, Rwanda; 26 St Paul’s Hospital Millennium Medical College, Addis Ababa, Ethiopia; 27 Hanagal Shri Kumareshwar Hospital and Research Centre Hospital, Bagalkot, India; 28 Department of Paediatrics, Maulana Azad Medical College, New Delhi, India; 29 Asiri Hospital Galle Queensbury, Karagitiva, Sri Lanka; 30 Clinical Sciences Division (CSD), Centre for Nutrition and Food Security (CNFS), International Centre for Diarrhoeal Disease Research, Dhaka, Bangladesh; 31 Emergency Department, Paediatric Emergency Medicine Leicester Academic (PEMLA) Group, Leicester, UK; 32 SAPPHIRE Group, Department of Health Sciences, University of Leicester, Leicester, UK; 33 Imperial College London, London, UK; 34 University Hospital of Wales, Cardiff, UK; 35 Sidra Medicine, Emergency Medicine, Doha, Qatar; 36 Emergency Department, Fakeeh University Hospital, Dubai, UAE; 37 Maroof Clinical Trial Unit, Research Department, Maroof International Hospital, Islamabad, Pakistan; 38 Emergency Department, HITEC-IMS, Taxila, Punjab, Pakistan; 39 Hospital Universitaria de mataro, Barcelona, Spain; 40 Department of Pediatrics, Northwest Clinics, Alkmaar & Den Helder, Netherlands; 41 Department of Pediatric Emergency Medicine, Geneva Children’s Hospital, Geneva University Hospitals, Geneva, Switzerland; 42 Emergency Department, Monash Medical Centre, Melbourne, Victoria, Australia; 43 Department of Paediatrics, School of Clinical Sciences, Monash University, Melbourne, Victoria, Australia; 44 Murdoch Children’s Research Institute, Leicester, Victoria, Australia

**Keywords:** Acute, severe acute asthma exacerbation Paediatric asthma, Core outcome set, Delphi survey, Consensus methods, Paediatric emergency medicine

## Abstract

**Abstract:**

**Introduction:**

Asthma is one of the most common chronic diseases of childhood and a leading cause of emergency department (ED) presentations worldwide. However, for those experiencing a severe exacerbation, there is a limited evidence base to decide which treatment works best. A major contributor to this, is the considerable heterogeneity in outcome selection which limits comparison between trials. The development of a core outcome set (COS) may improve this, enhancing the quality, comparability and applicability of future research. This protocol describes the development of the first internationally derived COS for trials evaluating interventions in severe, acute asthma exacerbations.

**Methods and analysis:**

A modified Delphi methodology will be used. An international steering committee comprising experts has been established through the Paediatric Emergency Research Network Asthma Working Group. For participants, this study will seek balanced representation across the six WHO regions and include clinicians who regularly manage children with severe acute asthma exacerbations and parents of children previously hospitalised with an asthma exacerbation. A target of 180 participants will be recruited, allowing for an anticipated 30% attrition rate between rounds and approximately 80 participants completing the final round. Three rounds of online surveys will be undertaken. Participants will rate the importance of outcomes identified through a systematic review and qualitative studies, with opportunities to suggest modifications and additional outcomes. Consensus after round 3 will be defined as either: (1) ≥70% of participants from both stakeholder groups scoring an outcome as critically important (7–9 on a 9-point Likert scale) and ≤15% scoring it as of limited importance (1–3) or (2) ≥90% of participants from either stakeholder group scoring the outcome as critically important.

**Ethics and dissemination:**

Ethical approval was granted by the Southern Adelaide Health Human Research Ethics Committee (HREC reference number 2025/HRE00375). Findings will be disseminated through peer-reviewed publications, conference presentations and stakeholder engagement activities.

This study has been prospectively registered with the Core Outcome Measures in Effectiveness Trials (COMET), registration number 1016.

**STRENGTHS AND LIMITATIONS OF THIS STUDY**

- This Delphi study is co-designed with both clinicians who regularly care for these children and by parents whose children have experienced an acute asthma exacerbation participating.
- All six WHO regions are represented in a balanced way giving broad representation across the globe allowing for participation from regions that have less well-established research networks.
- Given the time requirements of the Delphi study there is a risk that there may be loss of contributors/contribution over subsequent rounds; however, this has been mitigated by targeting an higher number of respondents in initial rounds.

## Introduction

 Asthma is a chronic inflammatory disease characterised by reversible airways obstruction and bronchospasm.^1^ It affects many children worldwide with an estimated 131 million children and adolescents living with asthma in 2023.^2^ In addition, it is one of the leading causes of emergency presentation worldwide with an estimated 700 000 annual visits per year in the USA.^3^ In low- and middle-income countries, there continues to be a disproportionately high rate of fatal outcomes for childhood asthma.^4^

Between one-third to a half of children who suffer from asthma will present with an exacerbation to an emergency department (ED) each year. While many of these children will respond to first line treatment a proportion will require escalated care. Escalated care can include one or more of the following: use of intravenous therapies, medications such as magnesium sulfate (MgSO_4_), aminophylline and salbutamol; and respiratory support such as high-flow nasal oxygen (HFNO) administration, non-invasive ventilation (NIV) and endotracheal intubation. A recent Australian study of more than 14 000 paediatric emergency presentations for acute asthma found that 7.3% (1003) children each year require such escalated care.^5^

However, there is a limited evidence base to decide which treatment, including which intravenous therapy will have the most benefit in the acute setting, and how to measure this. This is in large part due to the heterogeneity of outcome measures used in trials, making combination of results difficult.^6^ Our systematic review paper outlined this significant variation in the type, number and timing of outcome measures used, demonstrating 56 primary outcomes across 35 published studies. Overall response to clinical treatment was the most common primary outcome (47/56; 84%), with asthma scores (23/47; 49%), bedside pulmonary function tests (11/47; 23%) and change to vital signs (4/47; 8%) being the most used to assess response. In addition, multiple Cochrane reviews have concluded a lack of evidence base in this area is due in part to the inability to combine results from trials due to this heterogeneity.^6–9^ Addressing this has been identified as a priority by multiple national and international research networks.^10–12^ To address this issue the development and application of standardised sets of outcomes, also known as core outcome sets (COS), is required.

A process to develop COS has been outlined by the Core Outcomes Measures in Effectiveness Trials (COMET) initiative.^13^ This highlights the need to include both the people who are involved in the care of patients, usually clinicians and the people who experience the care, patients and their families. As a result of this initiative, multiple research groups have developed COS.^14^ In paediatric asthma, a group from the USA have published a COS for children with persistent asthma.^15^ This work highlighted the following outcomes: presence of interval symptoms, number of exacerbations and overall quality of life (QoL). However, there is no current consensus on outcome measure for use in clinical trials of children with acute, severe, acute asthma exacerbations.

As a measure to achieve this goal, the PERN (Paediatric Emergency Research Network) asthma working group was developed in 2017. PERN is a global research network incorporating paediatric research organisations from the USA, Canada, Europe, Spain, the UK, Ireland, Australia, New Zealand and Latin America.^16^ The inaugural publication from this asthma working group outlined the steps to achieve an agreed on set of COS for randomised controlled trials in acute, severe acute asthma exacerbation in children.^17^ These steps have mostly now been achieved including conducting a systematic review of outcomes measures utilised in trials of severe acute asthma exacerbation^6^ and global qualitative studies with both clinicians^18^ and parents of children.^19^ The final stage is to now conduct a three-round Delphi survey.

### Scope

We aim to reach a global consensus on a minimal set of core outcomes that should be measured and reported in all future clinical trials investigating the utility of escalated care for children with a severe acute exacerbation of asthma. This will include balanced representation across the six regions of the WHO from clinicians who regularly care for children with a severe acute exacerbation of asthma and parents of children who have received this care.

The Delphi will identify specific core outcomes, organised within broader outcome domains. An outcome domain related aspects of health or care, such as clinical response. A specific outcome describes what will be assessed, such as duration of supplemental oxygen therapy, whereas a measurement instrument describes how an outcome is assessed, such as an asthma severity score. Grouping outcomes into domains will support consideration of overlap and redundancy if a large number of outcomes meet the consensus criteria.

## Methods

### Study design

The study design was informed by the COMET COS Development Framework^20^ and will consist of four phases: (1) recruitment of a steering group with up to five representatives from each WHO region; (2) development of the initial survey which will be based on prior work including a systematic review^6^ and two qualitative studies.^18 19^ This initial survey will be reviewed for usability by survey experts and by parents who are on the steering group; (3) a three-stage online Delphi survey, with input from both the survey experts and parents and (4) an expert panel meeting, should it be required including clinicians and parent representation to ratify the final COS.

This study has been prospectively registered with the Core Outcome Measures in Effectiveness Trials (COMET), registration number 1016.

### Steering committee

The steering committee will be responsible for maintaining oversight for the overall conduct of the Delphi study and seeking adequate representation from each WHO region or country. This will also include parent representation and is anticipated to include at least two parents. All Steering Committee members and study contributors will be required to disclose any actual or potential conflicts of interest prior to commencement of the study and as they arise throughout the study duration. Any conflicts will be recorded and fully disclosed to the steering group with restrictions or withdrawal from study aspects if deemed necessary. To prevent result bias both clinician and parent steering group members will not participate in Delphi survey rounds.

### Development of outcome measures

A systematic review of outcomes measures and their measurement was performed by the lead author and reported treatment outcomes utilised in trials assessing intravenous therapy in severe acute asthma exacerbations.^6^ Together with two international qualitative studies that identified specific outcome measures considered important by clinicians^18^ and parents,^19^ this work has informed the development of the initial survey to be used in the first round of this Delphi study.

Response to clinical treatment was the most common primary outcome domain reported in our systematic review, with an asthma score being the most utilised outcome measure to assess this response, accounting for 40% of all primary outcomes. In addition, duration of oxygen therapy, total dose of beta_2_-agonist therapy and the need for second-line therapy featured high on the list of frequently used primary outcome measures. Finally, disposition and length of stay as outcome domains were also featured. These were both identified as important outcome domains by both clinicians and parents. Parents from around the globe identified the following outcome measures as important to include in trials: measure of work of breathing, side effects of treatment, costs of all treatment. An example of these candidate outcome domains and outcome measures is identified in table 1.

**Table 1:** Core outcome areas and examples of outcome measures for use in trials of severe acute asthma exacerbation

Outcome domains	Examples of specific outcomes
Clinical response and treatment requirements.	How long a child needs supplemental oxygen? Need for more medication after trial medication given
Length of stay	How long the child was in the emergency department?
Disposition	How many children were admitted to intensive care unit?
Social and financial	Total cost of all medication and tests? How long the child was away from school or normal activities?

### Stakeholders and recruitment

#### Parents

This study includes parents of children, aged between 2 and 17 years (up to 18th birthday), who have been treated for asthma and admitted to hospital or received treatment for severe acute asthma exacerbation in ED. Consideration has been given to under-representation from some WHO regions. To maximise global representation, recruitment will be supported through community partnerships, patient advocacy groups, family advisory councils, social media networks and established digital asthma communities used by patients and families. In addition clinicians will also, once necessary site-specific ethics is obtained, recruit patients seen in their daily practice. Potential participants who have further questions or who are interested in participating will contact a member of their region’s steering committee or the principal investigator or research co-ordinator by phone or email (provided in the Flyer or information sheet). It will be the responsibility of the steering group to identify adequate consumer representation, and to seek ethics if required to obtain representation.

For these respondents experiencing care, we will seek verbal consent to send them an information sheet/flyer. This will contain a QR code which will take them to the REDCap database which will outline the requirements for this study and seek their consent. This initial contact will be in the form of the ‘registration survey’, which will ensure that the respondents meet inclusion criteria and outlines the requirements of completing the survey and explains the language and concepts utilised in the survey. This ‘registration survey’ will be co-designed with parent members of the steering committee to maximise its approachability for participants.

The inclusion criteria are as follows: parents or caregivers of children aged 2 to 17 years (up to their 18th birthday) who received initial treatment for an acute wheezing or asthma exacerbation according to local asthma guidelines and who were subsequently admitted to hospital or treated in the ED as having a severe acute asthma exacerbation. Parents of preschool children presenting with wheeze for the first time may not yet have an established asthma diagnosis but are eligible if their episode was treated as an acute asthma exacerbation according to local guidelines.

This pragmatic eligibility criterion was intended to support meaningful family representation across healthcare settings. As only a small proportion of children presenting with an acute asthma exacerbation require escalated care^5^ (including intravenous bronchodilator therapy, non-invasive and invasive ventilation), requiring this treatment for eligibility would substantially restrict participation and potentially favour families from settings where intravenous therapies are more readily available. These criteria define eligibility for parent/caregiver participation; they do not constitute a standardised international definition of exacerbation severity.

#### Clinicians

Invitations to participate will be distributed via email through the partner networks that contribute to PERN, and to clinicians associated with the PERN asthma working group. They will communicate the aims of the study and expectations of participants. This will be done for each WHO region through the steering group. Paediatricians, paediatric respiratory specialists, emergency physicians and intensive care physicians will be eligible to participate if they regularly care for children with severe acute exacerbations of asthma and who indicated they will participate in the section on consensus on core outcomes. If a potential participant to this group has not provided care to a patient <18 years with a severe acute exacerbation of asthma in the preceding 6 months as defined by providing care which included one or more of the following: intravenous (IV) therapy, high-flow nasal oxygen (HFNO), continuous positive airway pressure, bilevel positive airway pressure (BiPAP) or intubation and mechanical ventilation, they will not be eligible to participate. Children in the preschool aged group, between 2 and ≤6 years old were included if they had been treated with any of the measures outlined meeting the criteria outlined above for severe acute exacerbation of asthma regardless of if they had an already established diagnosis of asthma.

### Participating countries and research groups

These will be identified from the international collaborative (PERN asthma working group and from other established research networks across the globe, for example, PERUKI (Paediatric Emergency Research in the United Kingdom and Ireland) and PREDICT (Paediatric Research in Emergency Departments International Collaborative in Australia and New Zealand). It is anticipated in some WHO regions such as the African, Eastern Mediterranean and South-East Asian regions, where formal paediatric research networks are not in place, there may be under representation. To promote geographical diversity within WHO regions, regional steering group members will support recruitment through local clinical and research contacts, including contacts outside established paediatric research networks. Recruitment will seek participation from multiple countries within each region. Given the anticipated challenges of securing sufficient participation across all six WHO regions, formal country-level quotas will not be imposed, as these could further restrict recruitment in settings with fewer established research connections. Participation will be reported by country, WHO region and stakeholder group to describe the representation achieved.

### Sample size

Decisions regarding sample size for Delphi studies are not typically based on statistical power. However, recent literature indicates that for multistakeholder Delphi studies that a sample size of 80 completed surveys in the final round of surveys achieves >80% replicability.^21^ Therefore, allowing for a 30% attrition rate^21^ per round we will have a target of approximately 180 which over the WHO regions gives 30 per region, this will include approximately 15 clinician and 15 parent participants. We aim to ensure that the final sample includes balanced representation from all key stakeholders (parents and clinicians) across all WHO regions.

### Delphi study

#### International online Delphi study

The Delphi method is an effective tool for reaching consensus in large group of stakeholders providing a structured, transparent and iterative method for achieving consensus among clinicians and patients. By conducting the Delphi process online, broad geographical participation will be facilitated, enabling accessibility. Iterative feedback between rounds will allow participants to reconsider their ratings among others within the group while promoting consensus agreement.

The overview of this process described subsequently is presented in figure 1.

**Figure 1:**
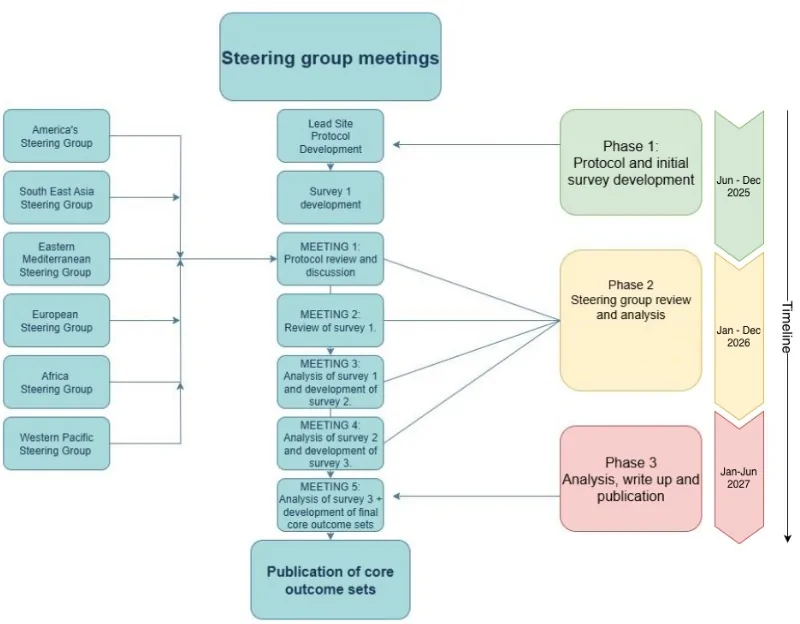
Study governance, stakeholder engagement framework and timeline for three round Delphi process.

#### Delphi round 1

In this first survey, respondents will be asked to rate the importance of a list of candidate outcome measures on a scale of 1–9, with 1 being not important at all to 9 which is extremely important. These will be categorised into outcome domains. The measures were collated from existing literature and reviewed for language and usability by consumers and survey experts (online supplemental appendix A for the survey instrument).

The participants will then have 3 weeks in which to submit their responses. A total of two reminders will be sent. Responses to the first Delphi round will be analysed by the steering group. No items will be removed. Open-ended responses will be collated, and those suggested by two or more respondents will be added to the list for the second survey. The steering group will meet regarding the results of the first round, analyse the data and determine the wording of additional questions to be administered during the next survey. This steering group may include consumers, dependent on their availability to ensure a balanced administration of survey results.

#### Delphi rounds 2

The second round of the Delphi survey will present all items from round one, as well as new open-ended responses. Participants will be provided with feedback including their previous rating and summary scores, ranked and presented as median from each stakeholder group. No responses will be excluded.

#### Delphi round 3

Items for round three will be based on the results of the second round, with inclusion if they are rated 7–9 (on the importance scale) by 70% or more participants and 1 to 3 by no more than 15% of participants in at least one stakeholder group. For those items that do not meet this pre-specified threshold they will not be carried to round 3 and will be reported as ‘no consensus’. Outcome ratings will be reported descriptively by WHO region, in addition to separately for clinicians and parents/caregivers. For each outcome, we will report the number of respondents and the proportions rating it as of limited importance (scores 1–3), important (4–6) or critically important (7–9). These will be exploratory in nature and will allow a description of regional variation and supplement the prespecified stakeholder-based consensus criteria, with cautious interpretation where regional respondent numbers are small.

### Consensus discussion

The core set of outcome measures from the Delphi survey will be selected after the third round of the survey according to the following criteria: 70% of participants scoring outcomes as 7 to 9 and 15% or less scoring 1 to 3 by both respondent groups; or 90% or more scoring 7 to 9 from either respondent group. The criterion of ≥90% of either stakeholder group scoring an outcome measure between 7 and 9 was included to ensure that outcomes considered essential by either clinicians or parents/caregivers were not excluded solely because of differences in priorities between stakeholder groups. There is precedence for this approach^20 22^ and is recommended in the COMET handbook.^13^ It is acknowledged that this approach may increase the final number of outcome measures included set and as such an online meeting may be conducted to reduce this. If there are responses that do not meet these prespecified criteria they will be carried over to the final meeting where they will undergo anonymous voting. If more than 12 outcomes meet the consensus criteria, a consensus meeting will be convened to prioritise outcomes and support feasible implementation. Outcomes will be considered within their respective domains to reduce overlap while preserving the breadth of the COS. Some selected outcome measures may require further work to establish how they should be measured.

These meetings will be offered to be online to facilitate international participation, and consideration will be given to different time zones. Invitations will be sent to all study steering group members and all round 3 respondents who select that they would like to be involved. The steering group members, as in the previous rounds, will not be eligible to vote. Following structured discussion, all round 3 respondents will undertake anonymous electronic voting. Outcomes for which at least 70% of attendees cast a vote that it should be included will be included in the final COS. Voting results will be presented in aggregate only and individual responses will remain confidential. If representation from both stakeholder groups and multiple WHO regions is insufficient to support consensus decision making we will hold the meeting with available participants, summarise the discussion and send all round 3 participants a final anonymous survey for voting on either the unresolved outcomes or for narrowing on the COS.

### Data collection and management

We will use the secure online survey platform Research Electronic Data Capture tools (REDCap)^23^ to administer the Delphi rounds over three rounds. Survey rounds will be delivered in English, Urdu, French, Chinese, Arabic and Spanish. Further languages will be considered based on respondents’ needs once all participants are recruited

### Ethics and dissemination

Ethics approval has been sought initially at Flinders Medical Centre, Adelaide, Australia. Ethics and governance approvals will vary depending on local requirements and governance. It will be the responsibility of the steering group member or nominated hospital lead to ensure adequate ethics approval prior to commencement.

### Consent

Many of the potential respondents of this study will be clinicians that will be able to make an informed decision in relation to participation. For both clinician and parent participants consent to participate from these respondents will be gained when they complete the registration form via the REDCap survey portal. For those parents that complete all three rounds of the Delphi study they will be eligible for a $A50 voucher. Distribution of this will be in accordance with local governance policies.

At the end of the project, the research team will make the results available to respondents via several different pathways. A summary of the results will be made available to the participants, via the PREDICT network’s website, following journal publication. The results will be published in a peer-reviewed journal and will also be presented at relevant conferences. The principal investigator holds the primary responsibility for publication of the result of the study, with co-investigators as co-authors. The order of authorship will be mutually agreed on prior to submission for publication. There are no publication restrictions. Approval must be sought from the principal investigator before any information can be passed onto a third party. We will not be using data linkage for this study.

### Data collection and confidentiality

Once separated from demographic data, participant responses will contain no identifying information, and the unique study identifier will be removed when the data are exported from REDCap. Responses will be aggregated so that the steering group reviewing them will not be able to link them to individual participants. Only the two investigators with REDCap administration access (chief investigator CG and research coordinator LA) will be able to link the responses to email addresses. This will occur only if temporarily required to support a participant experiencing difficulties completing the online survey. Data will be stored for 15 years in accordance with institutional and ethical requirements, in secure password-protected files on a server maintained by Monash University. All data generated in this study will remain confidential. No report, publication or presentation will contain any reference to individual participants, and all publications will be based on aggregated data.

### Limitations

Several limitations should be considered, although surveys will be translated into multiple languages, the study is coordinated primarily in English, which may introduce literacy and cultural bias. The online Delphi format may limit participation in settings with limited internet access or digital infrastructure. As participation is voluntary, parent and caregiver respondents may not be representative of the broader population, introducing the potential for self-selection bias. We recognise that country representation alone does not establish diversity in differing healthcare resources, treatment pathways or access to intensive care. These factors may limit the extent to which the resulting COS reflects perspectives across all settings within each region.

In addition, parent/caregiver eligibility relies on local clinical assessment and management pathways. Hospital admission does not necessarily indicate a severe acute asthma exacerbation, and differences in severity classification and admission thresholds may introduce heterogeneity in participants’ experiences. Also, we acknowledge that preschool wheeze is heterogeneous and may differ from asthma exacerbations in older children. The relevance of individual outcomes and the feasibility of particular measurement instruments may therefore vary with age. This study will seek a shared COS across the specified age range; it is not designed to establish separate age-specific COSs.

Finally, while stakeholder engagement is a strength of the study, steering group involvement in the development and review of survey content may introduce outcome bias. Adolescents were not included as a distinct stakeholder group in the Delphi process. As a result, outcomes prioritised by young people with lived experience of severe acute asthma exacerbations may have been under-represented and parent caregiver identified outcome measures should not be assumed to represent adolescents’ own priorities.

## Discussion

This co-designed COS study will follow robust methodology for its development and includes both clinicians and parents of children as important stakeholders from across the globe. It is anticipated that this will generate consensus on a COS. This will collectively enhance the design, conduct, and evaluation of future trials in this area. From this it is hoped that this will allow for generation of more robust clinical practice guidelines for use by clinicians who care for this cohort of children across the globe.

## Supplementary material

10.1136/bmjopen-2026-124611 online supplemental file 1

## Data Availability

No datasets have been generated or analysed at the protocol stage. Following completion of the study, de-identified Delphi survey data and consensus meeting outputs may be made available from the corresponding author on reasonable request, subject to approval by the study steering committee and relevant ethical and governance requirements.
