# Supplementary material for: Establishing a core outcome set for use in trials of severe acute paediatric asthma exacerbation (2–17 years): study protocol for an international co-design Delphi survey – a Paediatric Emergency Research Network study initiative
Source: BMJ Open. 2026 Oct 29;16(10):e124611. doi: 10.1136/bmjopen-2026-124611 (PMC13723850; doi:10.1136/bmjopen-2026-124611)
Supplement: Supplementary file 1 [file bmjopen-2026-124611.reviewer_comments.pdf]

PEER REVIEW HISTORY

BMJ Open publishes all reviews undertaken for accepted manuscripts. Reviewers are asked to complete a checklist review form and are provided with free text boxes to elaborate on their assessment. These free text comments are reproduced below.

ARTICLE DETAILS

Title (Provisional)

Establishing a core outcome set for use in trials of acute severe paediatric asthma exacerbations (2-17years): study protocol for an international co-design Delphi survey. A Pediatric Emergency Research Network study initiative."

Authors

Gray, Charmaine; Armit, Leah; Babl, Franz E; Powell, Colin; Yock-Corrales, Adriana; Santhanam, Indumathy; Johnson, Michael D; Dalziel, Stuart; Ong Yong-Kwang, Gene; Millar, Morgan M; Schuh, Suzanna; Irazuzta, Jose; González, Sebastián; Chebet Kimeto, Lilian; Hitayezu, Janvier; Bacha, Tigist; Yeshidinber, Abate; Vanaki, Raghavendra; Saxena, Romit; Gajaweera, Hasitha; Chisti, Md. Jobayer; Roland, Damian; Maconochie, Ian; Evans, Jordan; Alansari, Khalid; Ghanem, Abdullah; Waris, Saboora; Saeed, Ayeesha; Ramon Iglesias, Carlos; Borensztajn, Dorine; Siebert, Johan N.; Craig, Simon; Pediatric Emergency Research Networks (PERN), "On behalf of"

VERSION 1 - REVIEW

**Reviewer**	**1**
**Name**	**Takizawa, Takumi**
**Affiliation**	**Gunma University, Pediatrics, Graduate School of Medicine**
**Date**	**27-Aug-2026**
**COI**	**None**

This is a well-conceived protocol addressing an important methodological issue in pediatric acute asthma research. The development of an internationally applicable core outcome set is clearly needed, and the involvement of both clinicians and parents/caregivers is a strength. I have several comments, mainly regarding the definition of the target population and the proposed approach to international representation.

Major comments

1. Definition of acute severe asthma

The definition of “acute severe asthma” needs to be clarified. At present, eligibility appears to depend largely on local asthma guidelines and the judgement of the treating clinician as

to whether the episode was severe. For example, parents/caregivers are eligible if their child was treated according to local acute asthma guidelines and was admitted to hospital or treated in the emergency department as having severe asthma. This may result in considerable heterogeneity across countries and institutions, as thresholds for hospital admission, intravenous therapy, respiratory support, and intensive care differ widely. This is particularly relevant for a core outcome set intended for international trials.

The authors should provide a clearer operational definition of acute severe asthma, or at least specify the clinical or treatment-based features that define the target population. I would also suggest using "severe acute asthma exacerbation" consistently, where appropriate, to avoid confusion with "severe asthma" as a chronic disease severity category.

2. Inclusion of preschool children

The inclusion of children as young as 2 years also requires some clarification. The manuscript refers to children with an "acute wheezy or asthma exacerbation," which suggests that some preschool children without an established diagnosis of asthma may be included.

This is understandable from an emergency care perspective, but preschool wheeze is clinically heterogeneous and may not be equivalent to an asthma exacerbation in older children. The authors should clarify whether the proposed core outcome set is intended to apply equally to preschool wheeze and established asthma. It would also be useful to state whether age-related differences in the relevance of individual outcomes will be considered during the Delphi process.

3. Geographical representativeness

The aim of achieving balanced representation across all six WHO regions is a major strength. However, equal numbers from each WHO region do not necessarily ensure adequate geographical or healthcare-system diversity.

Several WHO regions include countries with very different healthcare resources, access to intensive care, treatment pathways, and thresholds for escalation of therapy. In addition, clinician recruitment will largely occur through PERN and other established research networks. The authors themselves acknowledge that some regions, particularly Africa, the Eastern Mediterranean and South-East Asia, may be under-represented because established pediatric research networks are less developed.

The manuscript would benefit from more detail on how diversity within each WHO region will be ensured. For example, will there be any attempt to limit over-representation from individual countries or research networks, or to ensure inclusion of both high-resource and lower-resource settings within each region?

4. How regional differences will be handled in the Delphi analysis

Related to the above point, it is not clear how geographical variation will be considered when determining consensus. The proposed consensus criteria distinguish between

clinicians and parents/caregivers, but not between WHO regions. Therefore, an outcome could potentially meet the overall consensus threshold despite being considered of relatively low importance in one or more regions.

Given that the study places considerable emphasis on balanced global representation, the authors should clarify whether outcome ratings will also be examined by WHO region, and how substantial regional disagreement will be handled. Even if regional analyses are exploratory rather than part of the formal consensus definition, this would be important to report.

Minor comments

1. The distinction between an outcome domain and a specific outcome measure is not always clear. For example, the manuscript discusses broad domains such as clinical response and length of stay, but also specific measures such as asthma scores, oxygen duration, ICU admission, and medication requirements. Please clarify whether the Delphi process is intended to identify core outcome domains, specific outcome measures, timing of measurement, or all of these.

2. Adolescents are not included as a separate stakeholder group, despite the target population extending to 17 years of age. The authors acknowledge this as a limitation. It would be useful to provide a little more justification for relying on parent/caregiver perspectives in this age group, as adolescents may prioritize some outcomes differently.

3. The recruitment strategy for parents/caregivers includes advocacy groups, family advisory councils, social media, and digital asthma communities. This is pragmatic, but it may preferentially recruit families with higher health literacy and greater engagement with healthcare or research. The authors already acknowledge self-selection and digital access as limitations; a brief description of how these biases will be minimized would strengthen the protocol.

4. The alternative consensus criterion, whereby an outcome can be included if ≥90% of either stakeholder group rates it as critically important, is reasonable in principle but could potentially increase the size of the final core outcome set. It would be helpful to provide a little more justification for this threshold and indicate whether it has been used in previous COS studies.

VERSION 1 - AUTHOR RESPONSE

Reviewer 1:
Comments to the Author

We thank the reviewer for these thoughtful comments. They have helped us clarify the target population, the scope of the core outcome set (COS), and how geographical variation will be reported.

Comment 1. Definition of acute severe asthma
The definition of "acute severe asthma" needs to be clarified. At present, eligibility appears to depend largely on local asthma guidelines and the judgement of the treating clinician as to whether the episode was severe. For example, parents/caregivers are eligible if their child was treated according to local acute asthma guidelines and was admitted to hospital or treated in the emergency department as having severe asthma. This may result in considerable heterogeneity across countries and institutions, as thresholds for hospital admission, intravenous therapy, respiratory support, and intensive care differ widely. This is particularly relevant for a core outcome set intended for international trials.
The authors should provide a clearer operational definition of acute severe asthma, or at least specify the clinical or treatment-based features that define the target population. I would also suggest using "severe acute asthma exacerbation" consistently, where appropriate, to avoid confusion with "severe asthma" as a chronic disease severity category.

Response:

We thank the reviewer for highlighting the need to clarify the operational definition.

During initial planning meetings, the co-authors supported this broader eligibility criterion to facilitate meaningful parent/caregiver representation.

We also acknowledge that admission alone does not establish exacerbation severity and that clinical assessments, treatment pathways and thresholds for escalation vary across countries and institutions. We have therefore clarified that these pragmatic criteria define eligibility for parent/caregiver participation rather than constitute a standardised international definition of severe acute asthma exacerbations. We have also acknowledged the resulting heterogeneity as a limitation and adopted "severe acute asthma exacerbation" consistently to distinguish the acute episode from chronic severe asthma.

The following section with regards to parent/caregiver selection has been adjusted to read as follows:

The inclusion criteria are as follows; parents or caregivers of children aged of 2 to17 years (up to their 18th birthday) who received initial treatment for an acute wheezing or asthma exacerbation according to local asthma guidelines and who were subsequently admitted to hospital or treated in the emergency department as having a severe acute asthma exacerbation.

This extra paragraph immediately following has been added:

This pragmatic eligibility criterion was intended to support meaningful family representation across healthcare settings. As only a small proportion of children presenting with an acute asthma exacerbation require escalated care[5] (including intravenous bronchodilator therapy, non-invasive and invasive ventilation), requiring this treatment for eligibility would substantially restrict participation and potentially favour families from settings where these therapies are more readily available. These criteria define eligibility for parent/caregiver participation; they do not constitute a standardised international definition of exacerbation severity.

And to the limitations section

Parent/caregiver eligibility relies on local clinical assessment and management pathways. Hospital admission does not necessarily indicate a severe acute asthma exacerbation, and differences in severity classification and admission thresholds may introduce heterogeneity in participants' experiences.

With the following clarified in the clinician recruitment section.

If a potential participant to this group has not provided care to a patient <18 years with a severe acute exacerbation of asthma in the preceding six months as defined by providing care which included one or more of the following: Intravenous (IV) therapy, High-Flow Nasal Oxygen (HFNO), Continuous Positive Airway Pressure (CPAP), Bilevel Positive Airway Pressure (BiPAP) or intubation and mechanical ventilation, they will not be eligible to participate.

Comment 2. Inclusion of preschool children

The inclusion of children as young as 2 years also requires some clarification. The manuscript refers to children with an "acute wheezy or asthma exacerbation," which suggests that some preschool children without an established diagnosis of asthma may be included.

This is understandable from an emergency care perspective, but preschool wheeze is clinically heterogeneous and may not be equivalent to an asthma exacerbation in older children. The authors should clarify whether the proposed core outcome set is intended to apply equally to preschool wheeze and established asthma. It would also be useful to state whether age-related differences in the relevance of individual outcomes will be considered during the Delphi process.

Response:

We agree that wheeze in preschool children is heterogeneous and that a diagnosis of asthma is not always established in this age group. We have clarified that the COS applies to preschool wheeze treated as asthma. We have added this distinction and its implications for interpretation to the manuscript. We will not claim that the Delphi establishes separate age-specific core sets and have added this to the limitation section.

In the clinician section as:

Children in the preschool aged group, between 2 -≤6years old were included if they had been treated with any of the measures outlined meeting the criteria outlined above for severe acute exacerbation of asthma regardless of if they had an already established diagnosis of asthma.

In the parent/caregiver sections as:

Parents of preschool children presenting with wheeze for the first time may not yet have an established asthma diagnosis but are eligible if their episode was treated as an acute asthma exacerbation according to local guidelines.

In addition to the limitations, we have added:

Also, we acknowledge that preschool wheeze is heterogeneous and may differ from asthma exacerbations in older children. The relevance of individual outcomes and the feasibility of particular measurement instruments may therefore vary with age. This study will seek a shared core outcome set across the specified age range; it is not designed to establish separate age-specific core outcome sets.

Comment 3. Geographical representativeness

The aim of achieving balanced representation across all six WHO regions is a major strength.

However, equal numbers from each WHO region do not necessarily ensure adequate geographical or healthcare-system diversity.
Several WHO regions include countries with very different healthcare resources, access to intensive care, treatment pathways, and thresholds for escalation of therapy. In addition, clinician recruitment will largely occur through PERN and other established research networks. The authors themselves acknowledge that some regions, particularly Africa, the Eastern Mediterranean and South-East Asia, may be under-represented because established pediatric research networks are less developed.
The manuscript would benefit from more detail on how diversity within each WHO region will be ensured. For example, will there be any attempt to limit over-representation from individual countries or research networks, or to ensure inclusion of both high-resource and lower-resource settings within each region?

Thank you for this comment. We agree that balanced representation across WHO regions does not necessarily ensure diversity within regions. Recruitment was supported by regional steering group members and their local clinical and research contacts, extending beyond established PERN networks. The recruitment record demonstrates participation from multiple countries within each WHO region. For example, clinicians completing registration included participants from Rwanda, South Africa, Kenya, Ghana and Ethiopia in the African Region; Pakistan, the United Arab Emirates, Qatar and Sudan in the Eastern Mediterranean Region; and India, Bangladesh and Sri Lanka in the South-East Asian Region.

This has been added to the methods section to aid clarity:

To promote geographical diversity within WHO regions, regional steering group members will support recruitment through local clinical and research contacts, including contacts outside established paediatric research networks. Recruitment will seek participation from multiple countries within each region. Given the anticipated challenges of securing sufficient participation across all six WHO regions, formal country-level quotas will not be imposed, as these could further restrict recruitment in settings with fewer established research connections. Participation will be reported by country, WHO region and stakeholder group to describe the representation achieved.

And this to the limitations section:

Country representation alone does not establish diversity in differing healthcare resources, treatment pathways or access to intensive care. These factors may limit the extent to which the resulting COS reflects perspectives across all settings within each region.

Major comment 4: Regional differences in the Delphi analysis

How regional differences will be handled in the Delphi analysis
Related to the above point, it is not clear how geographical variation will be considered when determining consensus. The proposed consensus criteria distinguish between clinicians and parents/caregivers, but not between WHO regions. Therefore, an outcome could potentially meet the overall consensus threshold despite being considered of relatively low importance in one or more regions.
Given that the study places considerable emphasis on balanced global representation, the authors should clarify whether outcome ratings will also be examined by WHO region, and how substantial regional disagreement will be handled. Even if regional analyses are exploratory rather than part of the formal consensus definition, this would be important to report.

Thank you for highlighting the distinction between global participation and regional agreement. We have added an additional comments to the methods section to allow for a regional analysis of outcomes by WHO regions to be conducted.

This section has been added under the Delphi round 3 section to aid clarity.

In addition, outcome ratings will be reported descriptively by WHO region, in addition to separately for clinicians and parents/caregivers. For each outcome, we will report the number of respondents and the proportions rating it as of limited importance (scores 1–3), important (4–6) or critically important (7–9). These will be exploratory in nature and will allow a description of regional variation and supplement the prespecified stakeholder-based consensus criteria, with cautious interpretation where regional respondent numbers are small.

Minor comment 1: Domains, measures and timing

The distinction between an outcome domain and a specific outcome measure is not always clear. For example, the manuscript discusses broad domains such as clinical response and length of stay, but also specific measures such as asthma scores, oxygen duration, ICU admission, and medication requirements. Please clarify whether the Delphi process is intended to identify core outcome domains, specific outcome measures, timing of measurement, or all of these.

Thank you for this comment. This Delphi aims to identify specific core outcomes within broader outcome domains. Grouping outcomes by domain also enables consideration of overlap and redundancy if many outcomes meet the consensus criteria.

The present Delphi focuses on identifying what should be measured. Selection of particular measurement instruments, such as a specific asthma score, and agreement on measurement timing will require further work. We have clarified these distinctions and revised the terminology throughout the manuscript.

The Delphi will identify specific core outcomes, organised within broader outcome domains. An outcome domain groups related aspects of health or care, such as clinical response. A specific outcome describes what will be assessed, such as duration of supplemental oxygen therapy, whereas a measurement instrument describes how an outcome is assessed, such as an asthma severity score. Grouping outcomes into domains will support consideration of overlap and redundancy if a large number of outcomes meet the consensus criteria.

And this section in the consensus discussion

If more than 12 outcomes meet the consensus criteria, a consensus meeting will be convened to prioritise outcomes and support feasible implementation. Outcomes will be considered within their respective domains to reduce overlap while preserving the breadth of the COS. Some selected outcome measures may require further work to aid in how they are measured.

Minor comment 2: Adolescent perspectives

Adolescents are not included as a separate stakeholder group, despite the target population extending to 17 years of age. The authors acknowledge this as a limitation. It would be useful to provide a little more justification for relying on parent/caregiver perspectives in this age group, as adolescents may prioritize some outcomes differently.

Thank you for this comment we acknowledge this is a limitation of the COS. We have amended the following wording to better reflect this.

As a result, outcomes prioritised by young people with lived experience of severe acute asthma exacerbations may have been under-represented and parent caregiver identified outcome measures should not be assumed to represent adolescent's own priorities

Minor comment 3: Parent/caregiver recruitment bias

The recruitment strategy for parents/caregivers includes advocacy groups, family advisory councils, social media, and digital asthma communities. This is pragmatic, but it may preferentially recruit families with higher health literacy and greater engagement with healthcare or research. The authors already acknowledge self-selection and digital access as limitations; a brief description of how these biases will be minimized would strengthen the protocol.

Thank you for this comment.

We have added the following to provide further clarity regarding our approach.

In addition clinicians will also, once necessary site specific ethics is obtained, recruit patients seen in their daily practice

Minor comment 4: Alternative 90% consensus criterion

The alternative consensus criterion, whereby an outcome can be included if ≥90% of either stakeholder group rates it as critically important, is reasonable in principle but could potentially increase the size of the final core outcome set. It would be helpful to provide a little more justification for this threshold and indicate whether it has been used in previous COS studies.

Thank you for this comment. The alternative criterion of ≥90% of either stakeholder group rating an outcome as critically important was included to ensure that an outcome considered overwhelmingly important by one stakeholder group is not excluded solely because it is assigned lower importance by the other group. This is particularly relevant to our study, which deliberately incorporates both clinician and parent perspectives.

This approach has precedence and is outlined in the COMET handbook. The following papers have been included and in addition to this the COS methodology paper has been referenced:

1. Knaapen M, Hall NJ, van der Lee JH, et al. Establishing a core outcome set for treatment of uncomplicated appendicitis in children: study protocol for an international Delphi survey. BMJ Open 2019;9:e028861. doi:10.1136 bmjopen-2018-028861

2. Wylde V, MacKichan F, Bruce J, Gooberman-Hill R. Assessment of chronic post-surgical pain after knee replacement: Development of a core outcome set. European journal of pain. 2015 May;19(5):611-20.

COS methodology paper

1. **Williamson PR, Altman DG, Bagley H, Barnes KL, Blazeby JM, Brookes ST, Clarke M, Gargon E, Gorst S, Harman N, Kirkham JJ. The COMET handbook: version 1.0. Trials. 2017 Jun 20;18(Suppl 3):280.**

The following has been added to the paper

There is precedence for this approach[22,23] and is recommended in the COMET handbook[24].

We acknowledge that our alternative criterion may therefore increase the number of outcome measures meeting consensus. To address this, our protocol pre-specifies that if the resultant COS is considered overly large (>12 outcomes), a consensus meeting will be convened to further prioritise outcomes within outcome domains, while preserving representation of outcomes considered important by either stakeholder group.

The following has been added to consensus discussion section

Is it acknowledged that this approach my increase the final number of outcome measures included set and as such an on-line meeting may be conducted to reduce this.

VERSION 2 - REVIEW

**Reviewer**	**1**
**Name**	**Takizawa, Takumi**
**Affiliation**	**Gunma University, Pediatrics, Graduate School of Medicine**
**Date**	**14-Oct-2026**
**COI**	

The authors have adequately addressed the reviewers’ requests.
