# Supplementary material for: Establishing a core outcome set for use in trials of severe acute paediatric asthma exacerbation (2–17 years): study protocol for an international co-design Delphi survey – a Paediatric Emergency Research Network study initiative
Source: BMJ Open. 2026 Oct 29;16(10):e124611. doi: 10.1136/bmjopen-2026-124611 (PMC13723850; doi:10.1136/bmjopen-2026-124611)
Supplement: Supplementary file 2 [file bmjopen-16-10-s001.docx]

**Appendix A**

**Survey 1. Draft of proposed survey for Round 1 of Delphi**
